# Deep Learning Based Methods for Breast Cancer Diagnosis: A Systematic Review and Future Direction

**DOI:** 10.3390/diagnostics13010161

**Published:** 2023-01-03

**Authors:** Maged Nasser, Umi Kalsom Yusof

**Affiliations:** School of Computer Sciences, Universiti Sains Malaysia, Gelugor 11800, Penang, Malaysia

**Keywords:** deep learning, artificial neural network, breast cancer, cancer detection, breast cancer diagnosis

## Abstract

Breast cancer is one of the precarious conditions that affect women, and a substantive cure has not yet been discovered for it. With the advent of Artificial intelligence (AI), recently, deep learning techniques have been used effectively in breast cancer detection, facilitating early diagnosis and therefore increasing the chances of patients’ survival. Compared to classical machine learning techniques, deep learning requires less human intervention for similar feature extraction. This study presents a systematic literature review on the deep learning-based methods for breast cancer detection that can guide practitioners and researchers in understanding the challenges and new trends in the field. Particularly, different deep learning-based methods for breast cancer detection are investigated, focusing on the genomics and histopathological imaging data. The study specifically adopts the Preferred Reporting Items for Systematic Reviews and Meta-Analyses (PRISMA), which offer a detailed analysis and synthesis of the published articles. Several studies were searched and gathered, and after the eligibility screening and quality evaluation, 98 articles were identified. The results of the review indicated that the Convolutional Neural Network (CNN) is the most accurate and extensively used model for breast cancer detection, and the accuracy metrics are the most popular method used for performance evaluation. Moreover, datasets utilized for breast cancer detection and the evaluation metrics are also studied. Finally, the challenges and future research direction in breast cancer detection based on deep learning models are also investigated to help researchers and practitioners acquire in-depth knowledge of and insight into the area.

## 1. Introduction

One of the common cancers identified globally among women is breast cancer, which has become the leading cause of death [1,2,3]. Based on the recent findings by the American cancer society, over 40,000 women and about 600 men died as a result of breast cancer disease [3]. There are four basic forms of breast cancer: benign, normal, in situ carcinoma and invasive carcinoma [1]. A benign tumor slightly alters the breast’s anatomy, it is not toxic and does not fit the description of dangerous cancer [4]. In situ carcinoma, on the other hand, only affects the system of mammary duct lobules and does not spread to other organs [5]. This kind of cancer is not very harmful and is treatable if detected early. The most severe type of breast cancer is invasive carcinoma, which has the potential to spread to all other organs [6]. Over many years, breast cancer can be identified through several methods such as mammography, X-ray, ultrasound (US), Portion Emission Tomography (PET), Computed Tomography, temperature measurement and Magnetic Resonance Imaging (MRI) [2,7,8]. Usually, the golden standard approach for breast cancer diagnosis is a pathological process. In order to maximize visibility, the extracted tissue is stained in the lab before being subjected to imaging analysis. The staining procedure frequently employs Hematoxylin and Eosin (H&E) [9]. In most cases, Histopathological image analysis and genomics can both be utilized to identify breast cancer [4,10]. A histopathological image is a microscopic picture of breast tissues and is very helpful in the early treatments of cancer [11]. The genomics field is primarily concerned with multi-scale connections between data on gene expression and medical imaging [4]. A more accurate diagnosis can be achieved with the use of radio-genomics [10]. In order to predict and identify cancer early, molecular analyses of tissues can be performed.

Recently, Computer-Aided Design (CAD) has been introduced [12,13] to simplify breast cancer identification. However, traditional computer-aided design systems generally depend on manually created features and therefore weaken the overall performance [13]. With the advent of machine learning and AI methods, deep learning-based techniques were recently studied for breast cancer detection [14,15]. Representation learning is the basis of deep learning techniques, which come from several layers. The representation is transformed from the lower to the higher levels at each end by combining the non-linear and simple modules, in which the lower-level features are more comprehensible and the higher-level features more abstract [14]. Compared to the ML methods [10,16], deep learning is more effective and requires fewer human interventions for the related pattern recognition schemes. This makes it capable of effectively solving complex problems in various areas such as image analysis [17], pattern recognition and natural language processing.

Following its successful applications in breast cancer detection, the number of research works on deep learning-based approaches has increased exponentially recently. This begs for a systematic review and summary of the existing works to help successive researchers and practitioners gain better insight into and understanding of the field. In the past, several literature reviews on breast cancer detection have been published. For instance, Yassin et al. [2] investigated the existing ML-based methods for breast cancer diagnosis. The authors comprehensively assessed different image modalities as well as the different ML-based classifiers used for breast cancer detection. The authors in [18] reviewed various deep learning-based methods for classifying breast cancer based on image processing. However, this work focuses on shallow feed-forward networks, while other deep learning-based methods were not emphasized. A study in [19] summarized recent studies that used deep learning (DL) methods to detect breast cancer disease based on different imaging approaches. The authors specifically focused on the three breast cancer imaging approaches, namely, MRI, mammography and ultrasound.

The authors in [20] examined several DL- and traditional ML-based methods for breast cancer prediction by reviewing a total of 8 papers and 27 papers in DL and ML, respectively. The authors discovered that most of the reviewed literature employed the imaging process; however, only a few of the reviewed articles applied genetics. The authors in [21] reviewed several imaging methods based on mammography for breast cancer diagnosis. Gupta et al. [22] presented a brief survey of different systems and methods for the early detection of breast cancer. In this study, various imaging methods which comprise radar-based imaging and microwave tomography were examined. Oyelade et al. [23] examined different deep learning-based methods for breast cancer diagnosis from digital mammography. Husaini et al. [15] examined the application of ML techniques and thermography for detecting breast cancer problems. In this method, various ML methods were investigated to process the breast cancer thermographic images.

Considering the above-mentioned several reviews on the deep learning-based methods for breast cancer detection, it could be seen that most of the existing review works particularly focus on the image-based methods for breast cancer detection problems. It can be seen that most of the existing studies emphasize the traditional ML-based approaches, while those focused on the deep learning-based techniques particularly covered very limited studies, with no clear comprehensive and systematic analysis of the existing approaches.

Therefore, this SLR aims to provide a comprehensive SLR to systematically analyze the existing literature in the area of deep learning techniques for breast cancer detection and to have more in-depth knowledge that is required for the early detection of breast cancer and proper treatments. The SLR is mainly focused on methods based on histopathological images and genomics. To provide more recent developments in breast cancer diagnosis, we consider studies conducted from 2010 to 2021. We also investigate the challenges and provide recommendations for future research to help researchers and practitioners in this area. The main contributions of this study are as follows:

- Summarizing the deep learning-based methods popularly applied for breast cancer detection.
- Identifying the deep learning-based methods with the best performances for breast cancer diagnosis.
- Investigating the datasets generally applied in the deep learning-based methods for breast cancer diagnosis.
- Summarizing the evaluation metrics used for breast cancer diagnosis using deep learning-based methods.
- Analyzing the research gaps and future direction for deep learning-based breast cancer detection.

The rest of the article is designed as follows: Section 2 describes the background of the deep learning-based methods. Section 3, Section 4 and Section 5 present the research methods, the research questions and the synthesis results. Finally, Section 6 and Section 7 summarize the limitations of the study and the conclusion of the paper.

## 2. Background of Deep Learning Methods

**Figure 1:**
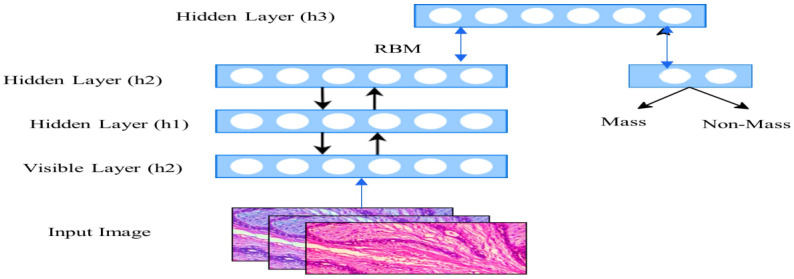
An illustration of the DBN for breast cancer detection.

Deep learning, in simple terms, is referred to as a machine learning method that employs learning representation to automatically determine feature representations from input data [11,24]. Unlike traditional learning (such as support vector machine (SVM), K-nearest neighbors (KNN), random forest (RF), etc.), deep learning does not need a human-engineered feature to optimally perform [10,12]. Several deep learning methods have been introduced in the past decades [25,26], which include the convolutional neural network (CNN), Restricted Boltzmann Machine (RBM), Recurrent Neural Network (RNN), Deep Autoencoder (AE), multi-layer perceptron and Generative Adversarial Network (GAN). These models have been applied and proved to be successful in several areas, including natural language processing [27], recommender systems [28], computer vision [29], medical imaging [30], etc. Brief explanations of these models are given in the following paragraphs:

RBMs: RBMs are generative deep learning-based methods that use blocks in a greedy, layer-by-layer form of network training and feature learning [31]. For the model to generate unbiased estimates of maximum likelihood learning, contrastive divergence (CD) is used during training. One of the popular examples of the RBM is the Deep Belief Network (DBN) [32]. By stacking many RBMs to data, the Deep Belief Network deep learning approach is learned in a greedy-wise, layer-by-layer style [33,34]. The top layer of the Deep Belief Network contains an undirected connection, which models the observed distribution between the hidden layer and vector space layer [13]. The lower layers of the Deep Belief Networks have direct connections. Similar to this, weight fine-tuning during training is carried out layer by layer via Contrastive Divergence (CD) [26]. Figure 1 illustrates the structure of the DBN technique.

**Figure 2:**
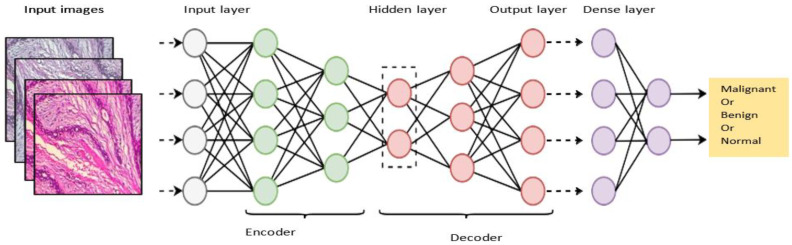
An illustration of the deep autoencoder.

Autoencoder (AE): The autoencoder technique replicates the copy of the input values as the output through the use of encoder and decoding units [35]. By reducing the dimensions of the data, autoencoder algorithms obtain the most discriminative features from unlabeled data [32]. In order to reduce error rates, the encoder converts the input data into hidden features that are further reconstituted using the decoder [36]. The method provided learning feature extraction techniques for avoiding handcrafted feature issues [35]. To generate a lower dimensional discriminative feature, the training of the auto-encoder is performed in such a way that the hidden layers are smaller than the inputs/outputs. [36]. A typical example of the autoencoder model is illustrated in Figure 2.

**Figure 3:**
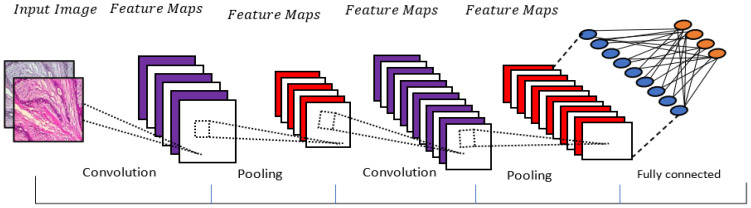
An illustration of the CNN model.

CNN: CNN is a neural network that has an interconnected structure. A CNN method is one of the popular deep learning methods that form convolutional operations on raw data [37]. It has been applied in various applications such as speech recognition, sentence modeling, image classification and, recently, medical imaging, including a breast cancer diagnosis. Basically, three layers make up the CNN: a convolutional layer, a pooling layer and a fully connected layer. These layers are stacked to create a deep architecture for automatically extracting the features [37]. Recently, several of the CNN models have been introduced by different researchers: VGG [38], AlexNet [39] and GoogleNet [40]. Figure 3 illustrates the structure of the CNN technique.

**Figure 4:**
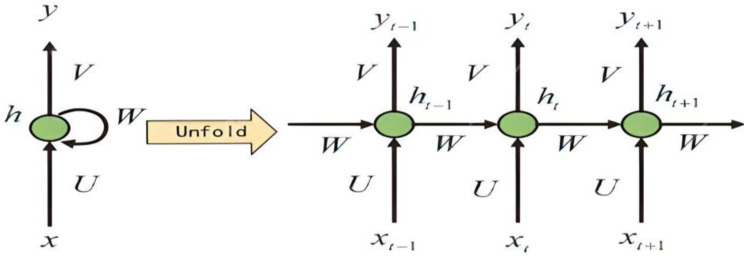
An illustration of the RNN model.

RNN: RNN is a supervised deep learning-based technique used for sequential data [41]. The hidden unit of the recurrent cell is used by RNN to learn complex changes by integrating a temporal layer for capturing sequential information [5]. Depending on the information accessible to the network, which is automatically updated to represent the network’s current state, the hidden unit cells may change [41]. However, the model suffers from vanishing or exploding gradients and is difficult to train, which limits its application for modeling temporal dependencies and longer sequences in a dataset [42]. To mitigate the issue of the vanishing and exploding gradients of RNN, new models such as Long Short-Term Memory (LSTM) and Gated Recurrent Unit (GRU) were introduced [36]. As a way to manage the flow of information into the network, LSTM included memory cells to store relevant data [34]. The LSTM can represent temporal dependencies and effectively capture the global features in sequential data to improve the speed [42]. However, one of the problems of the LSTM is the issue of the numerous parameters that are required to be updated in the course of the model training [34]. To reduce the parameter update, the GRU with fewer parameters, which makes it faster and less complicated, is introduced. The next hidden state’s updating process and contents exposure technique are different for LSTM and GRU [25]. While GRU updates the subsequent hidden states based on the correlation, subject to the amount of time required to maintain such information in the memory, the LSTM updates the hidden states using the summing operation. The basic structure of the RNN model is shown in Figure 4.

**Figure 5:**
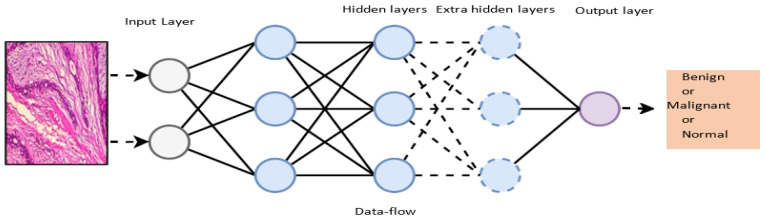
An illustration of the MLP.

Multilayer Perceptron (MLP): The MLP technique for feed-forward neural networks employs additional layers and nonlinear activation functions [43]. The simplest deep learning architecture is considered to be the multilayer perceptron [25]. It consists of a minimum of one hidden layer that is coupled in a feed-forward manner. For the most part, deep learning architectures are basically built using this framework [43]. The model’s linear approach can be changed into nonlinear models for neural performance using MLP. Consequently, they have been applied in different applications including natural language processing, a recommendation system and medical imaging. Figure 5 illustrates the structure of the MLP.

**Figure 6:**
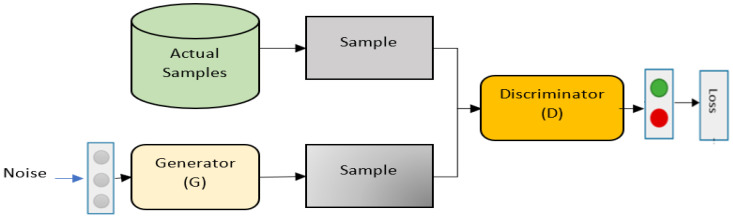
An illustration of the GAN.

Generative Adversarial Network (GAN): One recently introduced deep learning technique is the GAN, which was first discussed in [44]. For the maximum likelihood estimation technique, GAN offers an alternate approach. In a zero-sum game involving two neural networks, it uses both supervised and unsupervised learning techniques. It particularly aims to train a generative method that seeks to infer the distribution of the target data from the training data. Additionally, it makes use of the discriminative model, which provides an approximation of the likelihood that a sample of data is drawn from actual training data as opposed to the output. The learning rates and other parameters, such as the structure of the model, have a significant impact on how the GAN is trained. Numerous ad hoc methods are frequently needed to achieve effective convergence so as to increase the fidelity of the data generated. In addition, several extensions of the GAN methods were also devised to ease the complexity and improve the training process convergence. The Wasserstein Generative Adversarial Network and Loss Sensitive Generative Adversarial Network (LSGAN) are examples of this [45]. Research on the GAN is still in its early stages, though. Studies published recently suggested that GAN might be used for supervised learning applications. In the cases of recommender systems and information retrieval applications, using GAN’s unsupervised learning capabilities seems exciting [46]. An illustration of the GAN model is given in Figure 6.

The various deep learning techniques discussed above have made state-of-the-art methods in breast cancer detection. The main benefit of the deep learning-based method is its ability to automatically learn from unlabeled raw data. Such methods offer diverse capabilities for the detection of breast cancer. Table 1 summarizes the descriptions, weaknesses and strengths of each deep learning-based technique.

## 3. Methods

**Figure 7:**
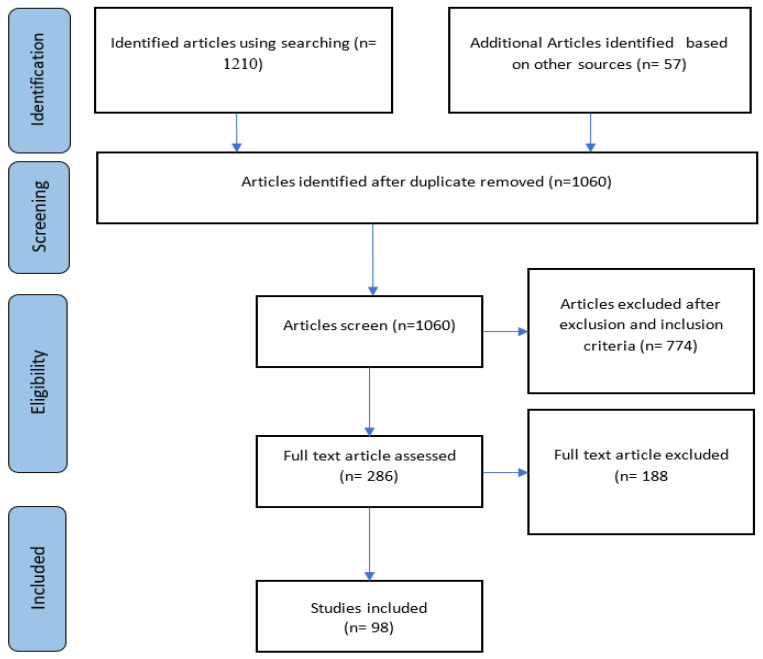
PRISMA method of the SLR.

This study employs the Preferred Reporting Items for Systematic Reviews and Meta-Analyses (PRISMA) guiding principles for conducting systematic reviews [47]. PRISMA provides a replicable and consistent method for identifying, selecting and critically examining the existing studies. It also offers direction for choosing, recognizing and evaluating the studies. Figure 7 displays the PRISMA process of the SLR. Details of the review process are given in the following subsection.

### 3.1. Data Sources and Search Strategy

**Figure 8:**
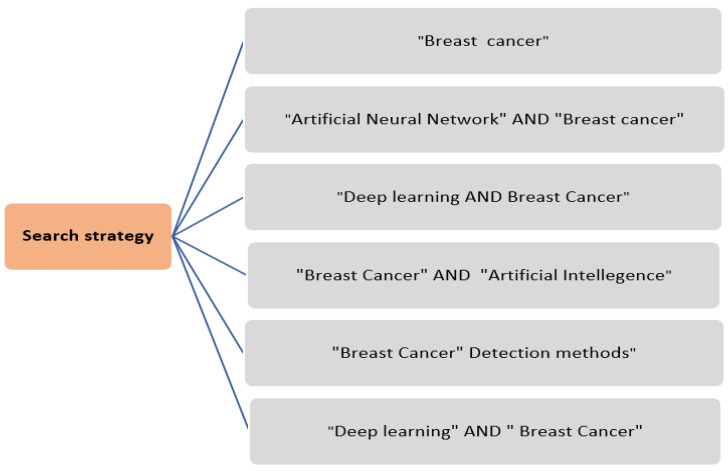
The search string used in the search strategy.

To gather more relevant studies, eight different bibliographic databases were considered for conducting the search process. The searched digital libraries include Scopus, Google Scholar, IEEE Xplore Library, Web of Science, SpringerLink, ScienceDirect, ACM Digital Library and PubMed. To obtain the latest and most comprehensive reviews, 2010 to 2021 was considered the timespan for the review. The search string comprised “breast cancer” or “cancer” or “((Artificial Neural Network)” or “((deep learning) AND (breast cancer))” AND (breast cancer))” or “((Artificial Intelligence) AND (breast cancer) AND (detection techniques))” or “breast cancer diagnosis”)). Figure 8 demonstrates the search strings used in the searching process.

### 3.2. Selection Criteria

In addition to the search string applied in the automatic search, we also conducted a manual search to comprehensively identify the relevant articles. In the identification stage, a total of 1267 studies were obtained from both automatic and manual searches. In the screening stage, 1060 published papers were chosen after filtering duplicate, unsuitable and irrelevant papers. A total of 774 articles were discarded after the removal criteria were applied to the remaining ones, and 286 articles were obtained. Finally, 98 research articles were designated for review after the quality assessment of the selected articles. Exclusively, papers utilizing genetic expression and imaging were included, and we narrowed down the number of publications by concentrating on journal and conference papers only. In addition to several kinds of gene expression and gene sequencing, the imaging modalities we took into consideration included ultrasound, magnetic resonance imaging (MRI), mammography and radiography. In this study, we concentrated on publications that employ deep learning-based approaches to implement the detection of breast cancer, as well as the publications that focused on breast cancer detection using both image and gene data. The exclusion and inclusion principles for the systematic reviews are stated in Table 2.

### 3.3. Quality Assessment

Analyzing the data contained in an SLR and evaluating the quality of the evidence it contains is equally significant. A bias resulting from a methodology may affect the results of a poorly conducted research work and therefore requires careful interpretation. Such articles must be explicitly excluded or at least identified as such in the systematic review. It is also necessary to select the correct criteria for assessing the quality of the evidence and any inherent biases in each study. To ensure the quality of the selected publications, the standard quality checklist questions (SCQ) designed in [48] are applied. To this end, following [49], we choose the articles that replied “yes” to at least seven questions. To ensure the findings significantly contribute to the review, the quality assessment will be taken into account alongside the data extraction [50]. Table 3 shows the SCQ used in the SLR.

### 3.4. Data Extraction and Synthesis

Firs, we took note of the key details, including the title of the paper, its publication year, an author list and the publisher. Afterward, we added some data for running the SLR, such as the deep learning method used, the reported accuracy and the evaluation metrics. The data synthesis stage particularly examines the associated findings from the data extraction process, which can be used to answer the research questions. After gathering the data, we visualize and analyzed the data through different visualization techniques and tools, such as a bar chart, a pie chart, histograms, etc.

## 4. Research Questions

Choosing RQs is very important in defining the overall purpose and expected outcomes of a study. Therefore, to achieve the main purpose of our SLR, we design the following RQs:

- RQ1: What are the most common deep learning-based methods applied for breast cancer detection?
- RQ2: What is the most effective deep learning-based method for breast cancer detection in terms of performance?
- RQ3: What are the commonly used performance evaluation metrics for deep learning-based breast cancer detection?
- RQ4: What are the common datasets used for deep learning-based breast cancer detection?
- RQ5: What are the challenges and future directions of the deep learning-based methods for cancer detection?

## 5. Results and Metanalysis

This section presents the metanalysis of the searched results of our SLR. It begins by presenting descriptions of the selected articles in this SLR and subsequently answers each of the RQs specified.

### 5.1. Description of the Selected Studies

**Figure 9:**
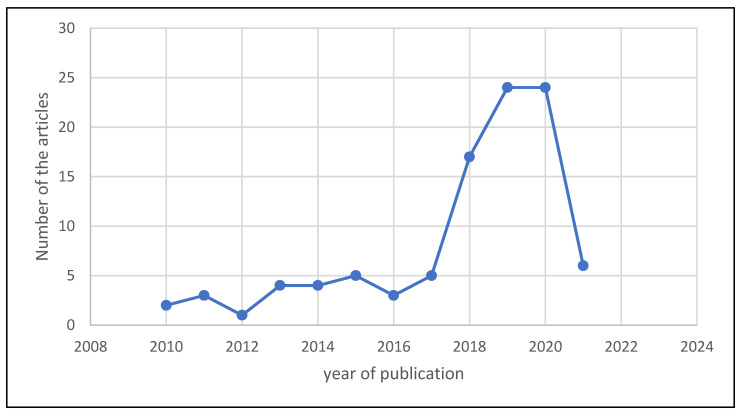
The number of papers with the corresponding years of publication.

**Figure 10:**
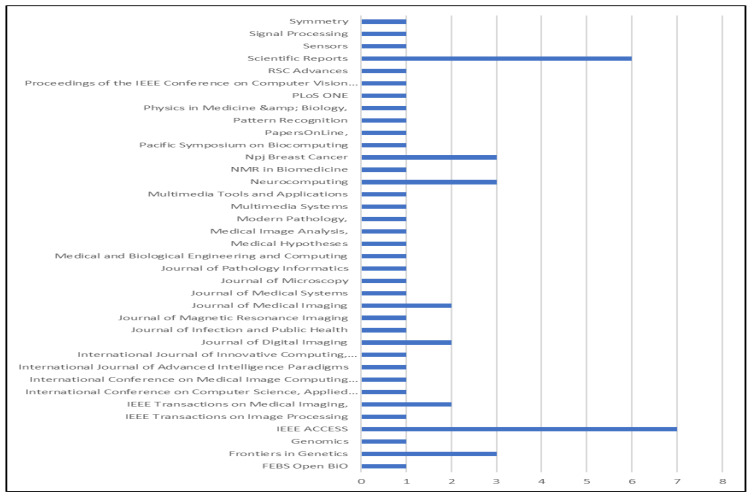
Number of articles per journal.

The number of publications regarding deep learning-based breast cancer detection methods from 2010 until 2021 is illustrated in Figure 9, which shows a chronological summary of the studies that were selected for this SLR. The illustration on the graph reveals a growing trend of studies in this field over recent years, especially from 2013 to 2015, when the number of published papers started to show a significant increase. The number of articles considered for the study mostly increased after 2013. Specifically, the years 2019 and 2020 saw the highest number of published articles (24) within a study year, followed by 16 papers in 2018 and 4 papers in 2021. As only 1 paper was examined in the year 2010, it can be seen that there was a decreased rate of relevant publications in that year. Figure 9 depicts the different journals and the total number of relevant articles used in the review. Similarly, the different names of the journals and the number of their corresponding papers used in the study are shown in Figure 10. It should be noted that, due to the space limit, only 70% of the reviewed articles and their corresponding journal names are shown in Figure 10.

### 5.2. RQ1: What Are the Common Deep Learning Methods for Breast Cancer?

To better provide effective cancer detection, a system needs to process  200 to 300 cells per frame, which is impossible through manual tracking [51]. Therefore, the development of effective technologies for breast cancer detection becomes necessary. In contrast, deep learning can be utilized to find patterns in unprocessed data. In recent times, deep learning is a common tool used to detect breast cancer. Deep learning methods have been demonstrated to be capable of diagnosing breast cancer up to 12 months earlier than those using conventional clinical procedures [16]. In addition, the techniques can be used to learn the most pertinent features to best tackle the issue. In recent times, different deep learning-based methods have been introduced for breast cancer diagnosis, which include CNN-, DNN-, RNN-, DBN- and AE-based approaches.

CNN is the most popular deep-learning technique that has been utilized in several studies for breast cancer detection [16]. The CNN is a deep learning model which represents hierarchical abstraction and consists of various layers which accept features as raw data [24]. The CNNs used for breast cancer diagnosis can be grouped into two: the transfer learning-based model and the de novo trained model [19]. The kinds of CNN models that were generated and trained from the scratch are referred to as “de novo models”. In contrast, the CNN techniques using previously trained neural networks such as AlexNet, residual neural network (ResNet), visual geometry group (VGG), etc. are called “transfer learning (TL)-based methods” [16]. Several methods used CNN-based methods for breast cancer diagnosis. The studies basically used the CNN model to extract various features based on the validated gene expression data in order to detect clinical outcomes in breast cancer [52,53]. Some authors use CNN to detect the mitosis process for the inversive breast cancer diagnosis based on histopathological imaging [54]. Others used the deep CNN method to classify and identify tumor-related stroma for breast cancer diagnosis [55,56,57,58]. In [59], a CNN-based method was utilized in combination with linear discriminative analysis and ridge regression based on image processing for breast cancer detection.

DNN has been shown to be effective for breast cancer detection [25,35,41]. The layers that make up a DNN include an output layer, a convolution layer, a fully connected layer and a pooling layer. Among these layers, a convolution layer is used for learning high-level characteristics. The purpose of a fully connected layer is to learn pixel-level features. The size of the convolved features can be decreased by a pooling layer, which lowers the amount of computation needed. This layer is capable of performing average pooling and maximum pooling operations [25]. Several DNN-based methods have been introduced for breast cancer detection. For example, Che et al. [60] used an attentive-based model for breast cancer diagnosis. The authors utilize multi-NNF DNN based on multi-modality information to improve the performances of breast cancer detection and prognosis. Lu et al. [61] applied a DNN technique for cancer detection. The authors used the deep learning-based feature representation of tumor-infiltrating lymphocytes in the cancer of the breast based on histopathological imaging for improving the detection process. Several other studies used the DNN model for detecting the subtypes of cancer by combining several types of transcriptomics data on breast cancer using deep learning techniques [62] and Identifying Differentially Expressed (DE) Biomarkers [63].

Another important deep learning-based method used for breast cancer detection is the RNN, which comprises many versions such as LSTM and GRU. RNN is a supervised deep learning method that is specifically used to process sequential data because it uses the loops and memories to keep track of previous computations when processing sequential inputs. Therefore, processing 3D volumetric images specifically as MRI image slices has been reported to be very helpful. Recently, several methods have applied the LSTM and GRU [64] for breast cancer detection. Some methods used the RNN technique for breast cancer detection. For example, a study in [64] introduced a gene-subcategory deep learning-based method that uses interaction-based learning for feature representations to improve the breast cancer subcategorical analysis based on gene expressions.

Autoencoders (AE) have also been applied for breast cancer detection [34]. A decoder rebuilds the images using the learned features to capture the essence of the raw features, and AE employs an encoder to properly transfer each image into a latent space. Several studies utilize AE for breast cancer detection. For example, Zhang et al. [65] used AE for improving deep learning for breast cancer detection. The authors employed Integrates Feature Selection and Feature Extraction to Predict the Clinical Outcome of Breast Cancer. In another study, Toğaçar, et al. [59] used the autoencoder-processed invasive breast cancer images to combine the CNN with linear discriminant analysis and ridge regression for breast cancer diagnosis. Xu et al. [66] introduced an approach based on histopathological images using a stacked sparse autoencoder (SSAE) to improve the model performances for breast cancer detection. A similar approach was introduced by Xu et al. [67], which used the SSAE model consisting of two SAEs for the detection of nuclei patches on breast cancer histopathology images to enhance breast cancer diagnosis.

GAN, which is a deep-learning-based generative model, has also been utilized for breast cancer detection. Shams et al. [68] designed a deep generative multi-tasking based on the combination of the GAN and CNN for reducing the death rate in breast cancer. The model uses strategies for achieving better accuracy in mammography diagnoses. Singh et al. [69] designed a GAN-based method for segmenting breast tumors inside the region of interest on a mammogram. The generative model creates a binary mask that defines the tumor region after learning to detect it. It motivates the generative network to produce binary masks that are as realistic as feasible. Additionally, GAN has been applied as image-augmentation methods recently to address the issue of limited data. Digital breast tomosynthesis data were used by the authors of [70] to detect anomalies and complete an image using GAN. The detection method described in this study produced encouraging results because it could locate suspicious areas without the need for training photos with anomalies. Fan et al. [70] used a generative adversarial technique with an improved deep network, bicubic interpolation and other techniques to create super-resolution images. GAN was employed by Guan and Loew [71] as a new mammographic image generator from the DDSM datasets, while CNN was utilized as GAN’s discriminator. Compared to other image-augmentation methods, GAN performed better.

DBN comprises a stack of RBMs, which are seen as the visible layer, and multiple hidden layers (the top two hidden layers contain random relationships), from which the deep features of the visible layer are extracted using a model of the generative probability union distribution [34,38]. Several studies used DBM for breast cancer detection; for example, Smolander et al. [33] introduced a deep learning-based approach for classifying gene expression data from complicated diseases by comparing DBN with SVM. Salma used a metaheuristic approach based on the DBN for breast cancer classification. Table 4 illustrates the deep learning-based methods used for breast cancer detection.

### 5.3. RQ2: Which Deep Learning Models Perform Most Effectively?

This section summarizes the performances of the different deep learning-based methods for cancer detection identified in this SLR. Table 5 demonstrates the summarized performances of different methods. Essentially, deep learning-based methods used two different approaches to classify breast cancer: multiclass and binary classification, which involve two instances and multiple subtypes, respectively. From the table, it can be seen that the use of the multiclass classification generally leads to lower performances compared to the binary classification. From Table 4, it can also be noticed that the majority of the used binary classifications performed better in most of the methods. In Table 5, the best accuracy for multiclass categorization was indicated to be 95.7% accuracy [52]. Regarding the task of classifying breast cancer subtypes, the author of the research essentially compared deep learning with machine learning. There have been numerous models employed, including CNN, DNN with the attention mechanism and many other DL techniques.

It should be noted that, because not all of the published works in the reviewed articles adopted the confusion matrix parameters, only the accuracy metrics were considered for analysis in the reviewed articles. Based on the results from Table 5, CNN-based techniques seem to be most popular for both multiclass and binary classification. Generally, it can be seen that a high performance is obtained when imaging data are used for breast cancer based on binary classification [83]. However, when it comes to subtypes classification (multiple classifications), genetic sequencing exhibits better results than imaging data. It can also be observed that, generally, the use of CNN results in outstanding performances for both gene expression and imaging data. An example of this is paper [83], which produced 99% accuracy for binary classification. The models used hybrid models that combine deep learning methods and machine learning as well as standalone deep learning models.

In conclusion, the studies made extensive use of different algorithms. However, in both gene sequence and image data, MLP and CNN were the most often utilized algorithms. The majority of the articles employed CNN and MLP with different parameters and properties; however, several other algorithms, including DNN, were also used.

### 5.4. RQ3: What Are the Evaluation Measures Commonly Used for Deep Learning-Based Breast Cancer Detection?

The evaluation metrics are essential for evaluating the models’ performances. However, deep learning methods are not rigorously evaluated using any specific set of metrics. Specifically, many performance evaluation metrics, which include recall, precision, F1-measure, accuracy, area under the curve (AUC), false-negative rate (FNR), etc., have recently been used by various researchers. We outline the evaluation metrics used in the reviewed papers based on the chosen publications in this section. The different performance measure formulas are displayed in Table 6.

The precision assesses the accuracy of the model’s positive predictions, whereas the accuracy indicates how many of the model’s overall predictions were correct. The recall of a classifier, sometimes referred to as sensitivity, is the proportion of positive cases that the classifier correctly detects. The ratio of successfully identified negative samples to all negative samples serves as a proxy for the classifier’s specificity. Although it makes sense to improve both precision and recall, there is an adverse relationship between the two metrics. By enforcing a higher precision, a lower recall may occur, and vice versa. This is often known as a trade-off for recall/precision measures. The harmonic mean of the recall and precision, which is referred to as the F-score, is a preferable metric to maximize. By adjusting the decision threshold value for a classifier, it is conceivable to observe the performance change in terms of the trade-off between particular metrics such as recall and precision. Parametric evaluation is the procedure of examining all confusion matrices that may emerge from altering the decision threshold.

The other metrics comprise precision and recall and the ROC curves. Classifiers can be compared based on the area under the ROC curve (AUC), with perfect models having an AUC value equal to 1 and fully random classifiers having an AUC equal to 0.5. The performance measure formulas, the references and the number of studies are shown in Table 5 for each metric. For the FPR and FNR, the lower the value of the performance metrics, the better the ability of models to generalize. The TP, FP, TN and FN in this table represent the true positive, false positive, true negative and false negative, respectively.

### 5.5. RQ4: What Datasets Are Available for Breast Cancer Diagnosis?

Generally, deep learning methods require a large amount of data for training the model and achieving improved performance. Consequently, a major barrier to using deep learning algorithms for medical diagnosis is the lack of data. Recently, several datasets have been published for breast cancer diagnosis. These datasets can either be private or public. The private datasets can be acquired through different academic-comprising universities across the world [118]. There were hardly any freely accessible public datasets. The accessible datasets for both imaging and gene sequencing are included in Table 7 and Table 8 under public and private. We discovered different public and private databases that contained gene expression data for healthy and sick individuals, even though gene expression data are less prevalent than imaging data.

Genome Atlas [119] includes the most popular datasets used for breast cancer analysis; it aims to detect the complete set of DNA changes in various kinds of cancer-related problems. It offers a large number of instances for researchers. Each participant’s clinical data are provided, along with some generic data. Understanding these modifications could aid the study into how various cancer types develop [57]. For a variety of cancer types, including breast cancer, the Genome Atlas dataset contains gene data. The METABRIC dataset, which comprises the clinical characteristics, SNP genotypes, CNV profiles and expression derived through breast cancers acquired from participants in the METABRIC study, is the second most widely used dataset [3]. Additionally, for the purpose of studying and researching cancer, NCI Genomic Data Commons (GDC) [120] offers scholars a vast collection of gene-related data. Genome and DNA sequencing information from studies on cancer detection can be found in the GEO database [118].

Compared to genetic data, imaging data are more readily available as datasets. The Wisconsin breast cancer (WBC) dataset, available from the UCI repository, is the most used imaging dataset [121]. It has features that were determined from a digital image of a breast mass which was sampled with a fine needle aspiration (FNA). A total of 11 attributes are listed for each of the 699 cases in this dataset. Clumps’ thickness, uniform cell size and shapes, marginal adhesion, the size of a single epithelial cell, the absence of mitoses, naked nuclei, bland chromatin, normal nucleoli and marginal adhesion is the independent properties.

Another popular breast cancer dataset for imaging is the DDSM dataset [122], which can be used on its own. CBIS-DDSM, an enhanced and condensed version of the DDSM dataset for the evaluation of breast cancer diagnosis, was just published by Clark et al. [123]. It includes improved ROI-segmented images in addition to a readily usable dataset. The dataset contains 891 mass cases and 753 microcalcification instances, respectively.

**Figure 11:**
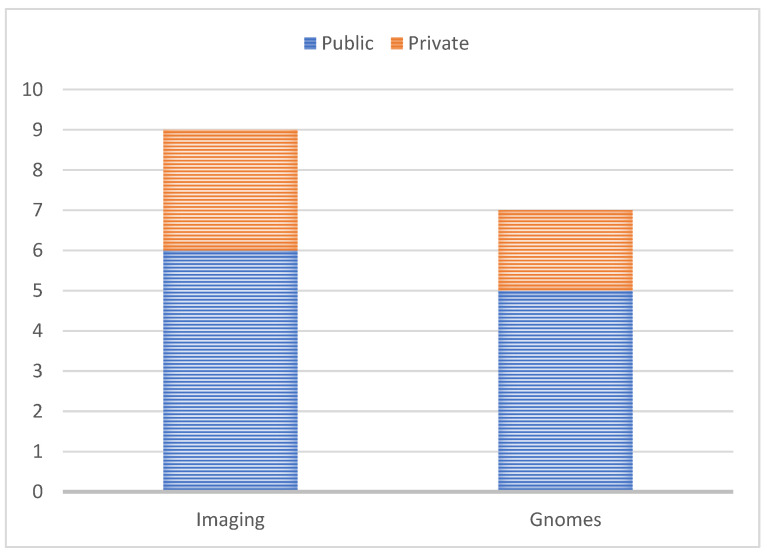
Distribution of the datasets for the cancer detection.

Another widely used dataset is the Mammographic Image Analysis Society Digital Mammogram Database (MIAS) [124]. The MIAS database contains 322 digitized MLO images and 161 cases with a variety of findings, including benign and malignant tumors as well as regular images. Another well-liked dataset for the detection of breast cancer is IN breast, which contains mammography pictures from cases involving screening, diagnosis and follow-up. The work’s most distinctive aspect is the expertly annotated ground truth that has been carefully associated with it. Table 7 and Table 8 summarize the datasets, and Figure 11 visualizes the distribution of the datasets for the cancer detection.

### 5.6. RQ5: What Are the Research Gaps, Challenges and Future Directions?

This section aims to answer RQ5 by presenting the research gaps and the future direction of deep learning-based methods for breast cancer detection. Although the existing studies have substantially contributed some basis for the deep learning-based methods for breast cancer detection, there are some gaps and future research directions in the field. The future research directions in this area and some of the important problems that have yet to be resolved are outlined as follows:

#### 5.6.1. Balanced Dataset

Deep learning methods have been demonstrated to be effective and promising in data mining. However, the evaluated datasets are virtually imbalanced for the deep learning-based method in the breast cancer area. First, there are not enough publicly available imaging and genomes databases that comprise the pathological heterogeneity and coexisting benign malignancy in different populations. Additionally, private datasets are arbitrary in terms of size, number and format. The work of annotation, which can be labor- and time-intensive, is another major difficulty for which clinical radiologists are not always available. An uneven breast cancer diagnosis is caused by an imbalanced training network dataset. In addition to creating adequate public breast cancer databases, future work will also focus on creating private datasets, which will primarily address issues with insufficient medical images, complex annotations and gene sequencing data. Moreover, to solve the problem of imbalanced data, some works applied oversampling methods [128,129]. However, it was shown in [130] that the oversampling method can lead to choice-based sample biases. Additionally, SMOTE (Synthetic Minority Oversampling Technique) was the only oversampling technique that was used [131]. This led to the conclusion that future studies might take into account using both under- and oversampling methods.

#### 5.6.2. Interpretable Deep Architecture

Designing deep models that are appropriate for the medical data would be challenging in addition to the complex radiological images. Hence, deep learning networks that are difficult to interpret pose a common problem in the detection of breast cancer. Along with diagnostic and histological reports, the ability of deep learning to handle heterogeneous datasets increases the possibilities for interpretable DL in breast cancer. Additionally, image captioning, which combines CV and NLP, has made significant progress [132]. The paradigm basically uses a standard encoder–decoder architecture to provide descriptions of given images based on RNN using visual attributes derived by CNN. The automatic creation of medical imaging reports appears in the medical domain, inspired by picture captioning [133]. This hybrid deep learning architecture can more accurately understand breast cancer diagnostic data because it uses text features to express radiomic features [134]. The first step is to pay attention to the learning model’s attributes and weights on the breast cancer images. The second is to allow for the understanding of the factors impacting the learning process involved within images of breast cancer.

#### 5.6.3. Clinical Application

For the management and treatment of patients, medical decisions are crucial. There is a need to put more emphasis on the deep learning-based method’s value in real-world applications in order to increase the accuracy of medical reports. Despite their best efforts, most studies did not fully utilize the relevant data that were available, despite the fact that they aimed to enhance the ultimate performance. There is a need to take breast cancer’s characteristics into account when using various imaging techniques (such as detailed malignant lesion features on ultrasound, hypoechogenicity, angular margin, posterior shadowing and internal vascularity). Additionally, there are several risk factors for breast cancer, such as age, family history and genetics, that are often taken into account by doctors but are overlooked in deep learning-based models. It can be another direction we can explore.

As a result, there is a need to pay closer attention to the deep features obtained using DLR, which might be used as information or data for additional research. In addition, multitask learning is a promising future direction. Through many tasks that gather the shared learning features one at a time, radionics features in a deep neural framework can reduce overfitting.

## 6. Limitations of the Study

In this SLR, various deep learning-based methods for breast cancer detection are identified. By developing our protocols, we aim to maximize both internal and external validity while addressing the RQs. There are still some restrictions and challenges to the validity of this argument, which can be encountered and explained in this section.

- This SLR is solely restricted to journal and conference materials that discuss breast cancer detection in DL. Several irrelevant research publications were found and eliminated from this review in the early stages of the study by using our search method. This guarantees that the chosen research papers met the requirements for the investigation. However, it is believed that incorporating other sources—such as extra sourcebooks, for instance—would have improved this review.
- We limited our search to items written in English. Due to the possibility of related publications in this area of study existing in other languages, this leads to linguistic bias. Thankfully, all of the papers gathered for this study were written in English. We are not language-biased as a result.
- Although the primary databases were considered when looking through the study articles, it is possible that other digital libraries with pertinent studies were disregarded. To overcome this limitation, we compared the search phrases and keywords to a well-known collection of research studies. However, when looking for the keywords, certain synonyms might be missed. To solve this issue, the SLR protocol has been updated to make sure no crucial phrases are omitted.

## 7. Conclusions

In this research work, we presented the SLR to systematically review the existing research works on breast cancer diagnosis based on the deep learning-based methods involving genetic sequencing or the histopathological imaging process. The SLR specifically adopts the PRISMA approach. Particularly, the applicability of various techniques in deep learning-based methods for breast cancer detection is studied. Several studies were searched and gathered, and after the eligibility screening and quality evaluation, 95 articles were identified. The results of the systematic review showed that the Convolutional Neural Network (CNN) is the most accurate and widely used model for breast cancer detection, and the accuracy metrics are the most popular method used for performance evaluation. Moreover, the datasets used for breast cancer diagnosis and the performance of different algorithms are also explored. Finally, the challenges and future research directions on breast cancer detection using deep learning techniques are also investigated to help researchers and practitioners acquire in-depth knowledge of and insight into the area. The widespread application of the CNN algorithms to data on both MRI images and gene expressions is a significant breakthrough. Comparing these models with other algorithms, they often produce positive results. It could be interesting for researchers to carry out additional research and apply more hybrid algorithms with CNN.

In addition, it was found that most of the studies on classifying images have not properly utilized the attention mechanism. This has provided researchers with the opportunity to employ attention mechanisms in the future to increase the precision of the deep learning methods. Given that this field is broad and that there is always the potential for more research and findings, researchers have recently been focusing on gene sequence data. Future researchers will have various chances to make a further contribution by combining different gene sequencing datasets to predict more results with larger datasets. More studies should emphasize deriving important aspects from gene expression data to improve outcomes and improve accuracy by employing confusion matrix parameters. This opens up the prospect for future studies to address a range of related issues, including determining risk levels and predicting the likelihood of recurrence. Future studies could focus on leveraging genetic data to create multiclass predictors, for example. Breast cancer detection and survival likelihood were the main focuses of the majority of studies, which only used genetic sequencing data with binary categorization. Furthermore, large-scale, thorough and fully labeled WSI datasets are currently lacking. Consequently, the creation of sizable public databases is crucial for future research.

## Figures and Tables

**Table 1:** Comparison of the deep learning techniques.

Techniques	Description	Weakness	Strength
DBN	Comprises two top-layer undirected connections and a directed connection at the lower layer.	The lengthy parameter initialization method makes it hard to train.	Able to extract hidden features from multiple data with great power.
DBM	Deep neural network with undirected connections between layers.	Due to the extensive parameters adjustment, the computation took a long time.	Enhances feature extraction using unsupervised training.
MLP	Models the data which have a simple correlation.	Delayed convergence and high complexity.	Nonlinear transformation.
AE	A neural network that has been trained to reconstruct the inputs under certain restrictions.	Lower ability to scale to high-dimensional data. Relies on high parameter tuning and numerical optimization.	Using an unsupervised learning strategy, it can learn more complicated feature representation.
CNN	Interconnected structural design motivated by the biological visual cortex.	Requires high parameterization tuning.	Highly effective for feature extraction with contextual data.
RBM	Undirected, bipartite graph with visible and hidden layers.	Although not tractable, the contrastive divergence can be utilized to learn the parameters.	Suitable for simple representation learning.
GAN	Deep neural network with a generator and descriminator.	Difficulty in convergence. Unstable learning process.	Appropriate for both supervised and unsupervised learning.

**Table 2:** Inclusion and exclusion.

Inclusion	Exclusion
Studies involving experimental results only	Studies without experimental results were not considered
Studies published from 2010 to 2021	Studies published before 2010 were excluded
Studies involving breast cancer detection only	Studies involving other cancer detections
Papers focusing on deep learning-based breast cancer detection	Papers focusing on other techniques used for breast cancer diagnosis
Studies written in English only	Studies written in other languages
Only journals and conferences are used	Other sources such as books, theses and magazines were excluded

**Table 3:** Quality Checklist.

No	Quality Question
SCQ1	Is the report clear and coherent?
SCQ2	Is the aim of the research clearly specified?
SCQ3	Is the data collection method obviously described?
SCQ4	Have the diversity contexts been well explored?
SCQ5	Are the findings of the study reliable?
SCQ6	Are there links between the data, interpretation and conclusion?
SCQ7	Are the methodology and experimentation process clear?
SCQ8	Are the research procedures documented adequately?
SCQ9	Are they important, if credible?
SCQ10	Could the research findings be replicated?

**Table 4:** The summary of the deep learning methods reviewed in this SLR.

Model	Description	References	Frequency
CNN	It uses convolution and pooling operations for feature extraction.	[52,53,54,55,56,57,58,59,61,72,73,74,75,76,77,78,79,80,81,82,83,84,85]	23
DNN	It is trained to construct posterior distributions for all possible values, using the input distribution’s encoding as a model distribution.	[60,61,62,63,86,87,88,89]	8
DBN	Includes directed connections at the lower layers and undirected connections at the two top layers.	[33,90]	2
RNN	An RNN version that incorporates a memory block to overcome the backpropagation problem.	[64]	1
MLP	A deep feed-forward network that produces a collection of outputs from a set of inputs.	[64,77,84,91,92,93,94,95,96,97,98,99,100]	13
AE	The generative model that recreates the input data in the output layer.	[59,65,66,67,101,102]	6
GAN	Deep learning technique that is generatively semi-supervised.	[68,69,70,71,103,104,105]	8

**Table 5:** Models used for gene sequencing data in selected papers.

DL Used	Brief Description of the Method	Classification	Method	Accuracy	Reference
FFNN	Uses negative and positive classes for the breast cancer diagnosis	Binary	Genomic	95%	[87]
FFNN	Uses negative and positive classes for the breast cancer detection	Binary	Genomic	92%	[106]
CNN	Provides breast cancer subtype classification based on the feature extraction	Multiclass	Genomic	95.6%	[52]
DNN	Uses miotic and non-miotic processes for identifying the breast cancer	Binary	Genomic	87%	[60]
DNN	Identifies risk categories based on the four classes	Multiclass	Genomic	94%	[86]
MLP	Axillary prediction of the lymph node status in breast cancer	Multiclass	Genomic	84%	[91]
FFNN	Breast cancer detection based on the cancer subtypes	Binary	Genomic	98.3%	[107]
CNN	Breast cancer detection based on the presence or absence of a tumor	Binary	Genomic	96.7%	[53]
CNN	Subtype identification based on the seven cancer types	Multiclass	Genomic	84.7%	[72]
AE	Predicts clinical outcomes of breast cancer	Binary	Genomic	84%	[65]
CNN	Breast cancer detection based on multiple categories of cancer	Multiclass	Genomic	76.4%	[73]
CNN	Mitotic and non-mitotic	Binary	Genomic	73.6%	[54]
CNN	Identification and classification of tumor-associated stroma in diagnostic breast biopsies.	Binary	Genomic	92%	[55]
CNN	Epithelial and stromal	Binary	Genomic	88%	[56]
DNN	Breast cancer molecular subtype classification	Multiclass	Genomic	87%	[108]
MLP	It uses the feature selection method for the detection	Binary	Genomic	98%	[109]
PNN	Breast cancer early diagnosis	Binary	Genomic	96%	[110]
MLP	Breast cancer prognosis detection	Binary	Genomic	96%	[93]
CNN	Breast cancer survival according to different features	Multiclass	Genomic	90%	[74]
CNN	Uses the feature selection method for the detection.	Binary	Genomic	98.7%	[59]
CNN	Breast cancer image classification based on epithelial and non-epithelial	Binary	Genomic	94%	[75]
DNN	Uses nuclei probability for the breast cancer tumor detection	Binary	Genomic	93%	[88]
MLP	Uses malignant/benign classification for the detection	Binary	Genomic	96.5%	[96]
CNN	Uses malignant/benign classification to determine the breast cancer	Binary	Genomic	94.5%	[76]
CNN	It determines the low risk and high risk for breast cancer	Binary	Genomic	98%	[58]
MLP	Uses multiclass based on the malignant, benign and other subclasses	Binary	Genomic	98%	[77]
MLP	Uses malignant/benign classification to determine the breast cancer	Binary	Genomic	97%	[97]
MLP	It uses the clustering method to identify breast cancer tumors	Binary	Genomic	96%	[98]
DBN	It uses multi-categories for the breast cancer gene classification	Binary	Genomic	90%	[33]
DNN	It uses the clustering method to identify breast cancer tumors	Binary	Genomic	84%	[89]
MLP	Identifies risk categories based on the four classes	Binary	Genomic	95%	[89]
FNN	Uses malignant/benign classification to determine the breast cancer	Binary	Genomic	94%	[90]
CNN	It identifies breast cancer lymph nodes	Binary	Genomic	84%	[79]
MLP	It identifies breast cancer tumors	Multiclass	Genomic	95%	[100]
CNN	It identifies breast cancer tumors	Binary	Genomic	93%	[80]
RNN	It identifies breast cancer tumors	Multiclass	Genomic	82%	[64]
CNN	Uses feature selection for the breast cancer detection	Multiclass	Genomic	94.5%	[81]
CNN	Uses feature selection for the breast cancer detection	Binary	Genomic	95.6%	[52]
DNN	It uses negative/positive classification to detect breast cancer tumors	Multiclass	Genomic	92%	[61]
CNN	Uses multiple categories based on A Luminal B HER2	Multiclass	Imaging	70%	[82]
CNN	Predicts breast tumors and responses to chemotherapy	Multiclass	Imaging	88%	[57]
CNN	Uses negative and positive to diagnose the breast cancer	Binary	Imaging	97%	[83]
MLP	Breast cancer subtype classification based on different categories	Multiclass	Imaging	90%	[84]
CNN	Uses negative and positive to diagnose the breast cancer	Binary	Imaging	71%	[85]
DBN	Uses the unsupervised method	Binary	Imaging	98%	[111]
GAN	Uses the generative unsupervised method for the breast cancer detection	Multiclass	Imaging	80%	[105]

**Table 6:** Performance evaluation measures.

Metrics	Description	Formula	References	Frequency
Accuracy	This is calculated by dividing the percentage of accurate predictions by the total number of predictions made by the model.	$\frac{\left(TN+TP\right)}{\left(TN+FN+FP+TP\right)}$	[33,52,53,54,56,57,59,60,61,62,65,72,73,74,75,76,77,79,81,82,87,88,89,90,91,93,95,97,98,100,106,107,108,109,110,112,113,114,115,116]	42
Precision	This is calculated by dividing the actual positive results by the true positive results.	$\frac{TP}{\left(TP+FP\right)}$	[54,59,60,61,62,72,73,75,76,77,79,80,81,88,89,90,96,97,100,109,114]	21
Recall/Sensibility	This is calculated as the ratio of actual positive samples that should have been discovered to true positive results.	$\frac{TP}{\left(TP+FN\right)}$	[33,54,59,60,61,62,72,75,79,80,81,89,90,91,96,97,100,109]	18
F1-score	This is measured as the model’s accuracy in each class.	$2\times \frac{Recall\times Precision}{\left(Recall+Precision\right)}$	[33,53,54,59,61,62,65,72,74,75,77,78, 80,81,88,89,90,91,96,97,100,109,117]	23
Specificity (TNR)	This is how well the prediction model predicts the percentage of negative tuples.	1−FP	[53,57,59,61,65,72,75,78,80,81,96,97,116,117]	14
AUC-ROC	It indicates the trade-off between the false positive rate (FPr) and the true positive rate (TPr).	Area under the ROC curve	[57,60,75,79,81,84,90,97,108,113,114,116,117]	13

**Table 7:** Genomic Datasets used for breast cancer diagnosis.

Datasets	Description	Links	Accessibility	Type	Instances	Reference
The Cancer Genome Atlas	It offers clinical data for every participant, along with some general data.	http://cancergenome.nih.gov	Public	Genomic	11429	[86]
METABRIC	Clinical characteristics, expression and SNP genotypes derived from breast cancers are included in the databases.	https://ega-archive.org/datasets/EGAD00010000268	Public	Genomic	543	[3]
Array Express Database	A database for high-throughput functional genomics data.	NA	Private	Genomic	NA	[125]
Geo Database	Data from high-throughput functional genomics investigations are stored in the functional genomics collection.	https://www.ncbi.nlm.nih.gov/geo/info/download.html	Public	Genomic	404	[118]
STRING and BIOGRID	A proteomic database involving the networks and interactions of proteins in a wide array of species.	NA	Private	Genomic	NA	[114]
GDC	It offers many gene-related data to researchers for use in the study and analysis of cancer.	https://gdc.cancer.gov/	Public	Genomic	9114	[120]
Spark Dataset	A public dataset which uses gene sequence data for breast cancer diagnosis.	https://drive.google.com/file/d/1yd1gwk2o	Public	Genomic	106	[112]

**Table 8:** Imaging-based datasets used for breast cancer diagnosis.

Datasets	Description	Links	Accessibility	Type	Instances	Reference
WBCD	It comprises 699 records from the FNA of human breast tissue. Each record has nine attributes.	https://archive.ics.uci.edu/ml/datasets/Breast+Cancer+Wisconsin+	Public	Imaging	569	[121]
Helsinki University	A customized private dataset designed by the Cancer Institute and Helsinki University.	NA	Private	Imaging	NA	[56]
MRI Data	The datasets are collected provisionally under an HIPAA-compliant approval from the institutional board.	https://wiki.cancerimagingarchive.net/display//Public/RIDER+Breast+MRI#2251275749b786f1af5747c39abd8eda0d12e2b7	Public	Imaging	1500	[6]
University of Vermont Medical Center	A customized private dataset designed by the University of Vermont Medical Center.	NA	Private	Imaging	NA	[9]
DDSM	It is a combination of digitalized benign without callback, benign, normal and cancer volumes that were carefully chosen.	https://wiki.cancerimagingarchive.net/display/Public/CBIS-DDSM#2251662	Public	Imaging	10239	[122]
Stanford Tissue Microarray Database	A database that offers tools for tissue microarrays, design, image scoring and annotation to researchers.	NA	Private	Imaging	-NA	[126]
MIAS	It is available on a 2.3 GB 8 mm (Exabyte) tape and has 322 digitized films.	http://peipa.essex.ac.uk/benchmark/databases/index.html	Public	Imaging	322	[124]
IN breast	It consists of images captured between 2008 and 2010 at the Breast Center in CHSJ, Porto.	http://medicalresearch.inescporto.pt/breastresearch/index.php/Get_INbreast_Database	Public	Imaging	410	[127]
CBIS-DDSM	It provides better ROI-segmented images in addition to a readily useable dataset.	https://wiki.cancerimagingarchive.net/display/Public/CBIS-DDSM	Public	Imaging	1644	[123]

## Data Availability

Not applicable.
